# Potential Therapeutic Targets for PPAR*γ* after Spinal Cord Injury

**DOI:** 10.1155/2008/517162

**Published:** 2008-03-27

**Authors:** Dana M. McTigue

**Affiliations:** Department of Neuroscience and Center for Brain and Spinal Cord Repair, The Ohio State University, Columbus, OH 43210, USA

## Abstract

Traumatic injury to the spinal cord results in multiple anatomical, physiological, and functional deficits as a result of local neuronal and glial cell death as well as loss of descending and ascending axons traversing the injury site. The many different mechanisms thought to contribute to protracted secondary cell death and dysfunction after spinal cord injury (SCI) are potential therapeutic targets. Agents that bind and activate the transcription factor peroxisome proliferator-activated receptor-*γ* (PPAR-*γ*) show great promise for minimizing or preventing these deleterious cascades in other models of CNS disorders. This review will summarize the major secondary injury cascades occurring after SCI and discuss data from experimental CNS injury and disease models showing the exciting potential for PPAR*γ* therapies after SCI.

## 1. WHAT IS PPAR*γ*?

“PPAR” is an acronym for peroxisome proliferator-activated receptor, which refers to a family of nuclear hormone receptors that function as ligand-activated transcription factors. Three PPAR isoforms have been identified to date, including PPAR*α*, PPAR*β*/*δ*, and PPAR*γ* (for review, see [1–3]). Upon activation, PPAR molecules heterodimerize with retinoid X receptors (RXRs), which are the nuclear receptors for 9-*cis* retinoic acid. After dimerization, the PPAR/RXR complex binds to specific promoter sequences on target genes where it can promote or repress gene activation. Most initial research on these molecules focused on their role in lipid metabolism and homeostasis [1, 4]. However, it is now known that PPARs function in a much broader array of physiological functions, both under normal conditions and following injury or disease. In particular, upregulation of PPAR*γ* mRNA has been detected in inflammatory cells and in experimental models of CNS injury such as ischemic stroke [5, 6]. PPAR*γ* agonists appear to have potent anti-inflammatory and neuroprotective actions [7–9]; thus, this transcription factor may be involved in coordinating cellular responses to CNS injury. This also presents the opportunity to enhance neuroprotection by leveraging PPAR*γ* expression through administration of specific agonists following CNS damage. Indeed, over the past decade, several studies have revealed beneficial actions of promoting PPAR*γ* activation in experimental models of CNS injury, ischemia, and disease. Less work has examined the potential of promoting PPAR*γ* activation following injury to the spinal cord, for which current clinical therapies are limited. This review will summarize the documented beneficial actions of PPAR*γ* following CNS injury and illustrate how they may also promote anatomical and behavioral recovery after spinal cord injury (SCI).

## 2. SPINAL CORD INJURY: THE FACTS

In the United States, a new SCI is sustained on average every 41 minutes, which results in *∼*1100 new cases each year. The majority of these injuries are caused by motor vehicle accidents, followed by accidents such as falling from ladders or diving into shallow water [10]. Most SCI's occur in young individuals, particularly males—in their late teens or twenties. Because medical care has improved dramatically during the previous century, most individuals can expect to live many years following an SCI. Their lives, however, are not easy and they have many issues with which to deal on a daily basis. In the eyes of most uninjured people, the most obvious problem affecting SCI individuals is their inability to walk. While this is clearly a significant obstacle to overcome, a recent survey of paraplegics and quadriplegics revealed that regaining locomotor function is actually of lesser importance to them compared to the many other issues they face [11]. For instance, quadriplegics would prefer restoration of hand and arm function over walking. Paraplegics' top choice would be regaining normal sexual function, an important issue in terms of relationships with significant others, and the ability to have a family. Also high on the list for all spinal-injured people was return of bowel and bladder function. Other serious issues many face include potentially fatal autonomic dysreflexia, pressure sores that can take several months to heal, and untreatable intractable pain [10].

Since it is impossible to prevent the occurrence of most SCIs, our best hope is to improve the level of recovery achievable after an SCI occurs. Most SCIs result from a contusion-type injury in which the vertebral bodies and/or intervertebral discs are rapidly displaced into the spinal canal causing crushing and bruising of the delicate spinal tissue [12, 13]. The initial impact leads to immediate hemorrhage and rapid cell death at the impact site. This is followed by multiple secondary injury cascades that cause further tissue loss and dysfunction [10, 14]. If these secondary injury processes were minimized or eliminated, the outcome for patients would be greatly improved. Many of these cascades are potential targets for intervention by activation of the transcription factor PPAR*γ*. Indeed, two recent studies demonstrated that treatment of SCI rodents with a PPAR*γ* agonist results in significantly improved anatomical sparing and locomotor abilities [15, 16]. The rest of this review will discuss specific secondary injury processes that occur after SCI and how PPAR*γ* activation may be used to lessen their impact.

## 3. GLUTAMATE EXCITOTOXICITY: A GOOD TRANSMITTER GONE BAD

Within minutes of SCI, extracellular glutamate levels rise within and around the injury site [17]. This potent neurotransmitter can then diffuse to surviving cells, bind to surface receptors, and lead to what is known as excitotoxic cell death [18]. Especially vulnerable to excitotoxicity are neurons and oligodendrocytes, which express a full complement of glutamate receptors. Loss of neurons at the injury site will lead to direct denervation and paralysis of muscle fibers innervated by those neurons, thereby contributing to motor deficits. Because a significant amount of sensory processing occurs within the spinal cord, especially that involved in pain and temperature sensation, loss of neurons can also lead to hypersensitivity, paresthesia, enhanced and prolonged pain, and/or total loss of pain and temperature sensation. Excitotoxic injury to oligodendrocytes can result in demyelination of axons around the injury site. This, in turn, will lead to a drastic reduction or complete halt of axonal transmission, thereby enhancing the disconnection between the brain and spinal segments below the level of injury. Thus, excitotoxicity has the potential to markedly exacerbate the functional problems encountered after SCI. Indeed, the involvement of excess glutamate in cell death after SCI was demonstrated by studies in which early treatment with glutamate antagonists significantly enhanced tissue preservation and functional recovery following SCI in rats [19, 20].

A major mechanism responsible for maintaining low extracellular glutamate levels is astrocytic uptake via glutamate transporters, including GLT1/EAAT2 which is responsible for removal of up to 90% of extracellular glutamate. While glutamate transporters are effective under basal conditions, they become saturated when glutamate levels rise substantially above normal. Thus, a mechanism for increasing expression of GLT1/EAAT2 and other glutamate transporters could be highly beneficial after SCI. Recent work reveals that PPAR*γ* activation may do just that. Using a cell culture model of ischemic preconditioning, Romera et al. [21] showed that preconditioning upregulates PPAR*γ* expression in neurons and astrocytes, and that treatment of the cultures with a PPAR*γ* agonist significantly increased astrocytic expression of GLT1/EAAT2 mRNA and protein. They also showed that this increased expression translated into enhanced glutamate uptake and reduced cell death. The proposed mechanism was a direct increase in EAAT2 promoter activity induced by activated PPAR*γ*. A direct neuroprotective action by PPAR*γ* activation under excitotoxic conditions has also been demonstrated using cultures of pure cortical neurons [22]. In vivo evidence supports the notion that PPAR*γ* activation is protective against glutamate excitotoxicity. For instance, treatment with a PPAR*γ* agonist decreased neuron loss caused by intracortical injection of a glutamate receptor agonist [22]. While changes in glutamate levels in SCI models treated with PPAR*γ* agonists have not yet been measured, protection against glutamate excitotoxicity is a plausible mechanism by which PPAR*γ* could improve outcome after SCI.

## 4. LIPID PEROXIDATION

A well-documented pathological process occurring early after SCI is the formation of reactive oxygen and nitrogen species (ROS and RNS, resp.); this results from increased intracellular calcium levels, mitochondrial dysfunction, arachidonic acid breakdown, and activation of inducible nitric oxide synthase (iNOS) [23–25]. Initially thought to be a problem only in acute SCI tissue, newer studies have revealed that indices of free radical damage are present throughout the first week after injury [26, 27]. ROS and RNS cause lipid peroxidation as well as oxidative and nitrative damage to proteins and nucleic acids [27, 28]. In addition, oxidative damage exacerbates mitochondrial dysfunction [29] and contributes to intracellular calcium overload which activates proteases resulting in breakdown of cytoskeletal proteins [27, 30]. Thus, the collective damage induced by ROS and RNS is far-reaching and likely contributes to cellular death and functional loss after SCI.

PPAR*γ* activation after SCI could dampen the damage induced by ROS and RNS in multiple ways. First, PPAR*γ* activation may reduce the overall level of free radicals present in the injured tissue since PPAR*γ* activation leads to decreased nitric oxide, cyclooxygenase-2 (COX-2), iNOS, and nitrotyrosine levels in animal models of amyotrophic lateral sclerosis (ALS), multiple sclerosis (MS), ischemia, and neuroinflammation [31–37]. In addition, PPAR*γ* agonists may increase the levels of antioxidants in or around the injured tissue. For instance, catalase levels were elevated by PPAR*γ* agonist treatment following intracerebral hemorrhage [38]; with increased antioxidant levels, the surviving tissue will be better equipped to fend off assault by free radicals. Thus, agonists that stimulate PPAR*γ* may reduce the levels of free radicals and at the same time, elevate enzymes essential for combating free radicals that remain. This in turn would reduce the number of neurons and glial cells that die in the subacute phase of SCI. Studies by our group and others have shown that treatment with the PPAR*γ* agonist pioglitazone resulted in an increase in the number of motor neurons spared after SCI, which might have been due, at least in part, to a reduction in post-SCI oxidative damage [15, 16]. By promoting motor neuron survival in human SCI, significant preservation of segmental function may be possible. Although complete recovery of “normal” function may not be feasible, even partial recovery of hand function, for instance, could drastically improve the quality of life for an individual with SCI.

## 5. INFLAMMATORY-MEDIATED CELL DEATH

A well-characterized event after spinal trauma is local microglial activation, inflammatory cell infiltration, and upregulation of proinflammatory mediators. Indeed, several studies have shown the rapid rise in proinflammatory cytokines and chemokines which stimulate inflammatory cell infiltration into the injured spinal cord [14]. Once present within the damaged and surrounding parenchyma, inflammatory cells such as neutrophils, macrophages, and lymphocytes can exacerbate tissue damage. For instance, activated macrophages and microglia produce cytotoxic molecules such as iNOS, TNF*α*, IL-1*β*, and IL-6. Interestingly, PPAR*γ* levels are upregulated in activated microglia and macrophages [35, 39, 40] and activation of PPAR*γ* in these cells can decrease production of proinflammatory mediators [40–42]. The mechanisms contributing to these effects include antagonism of AP-1, STAT, and NF*κ*B levels as well as concomitant increases in I*κ*B*α* levels. Collectively, these actions will reduce the inflammatory potential of the treated cells [37, 38, 40, 43]. Accordingly, PPAR-induced inhibition of microglia/macrophage accumulation and release of proinflammatory cytokines has been detected in animal models of Alzheimer's disease, Parkinson's disease, and MS [33, 34, 44–46]. PPAR*γ* activation can also reduce the differentiation of monocytes into macrophages [45], promote macrophage apoptosis [47], and decrease T-cell proliferation, which collectively would result in reduced numbers of infiltrating inflammatory cells into the injured spinal cord [48]. Indeed, PPAR*γ*-induced reductions in neutrophil, T-cell, and macrophage infiltration have been shown in animal studies of experimental allergic encephalomyelitis (EAE, an animal model of MS) and intracerebral hemorrhage [33, 38, 48]. This may be due, in part to, a PPAR*γ*-mediated reduction in chemokines, which elicit inflammatory cell recruitment to the CNS. For instance, mice with EAE given oral PPAR*γ* agonists expressed lower levels of MIP1*α* and RANTES within the brain compared to control mice [33].

Collectively, these data suggest that PPAR*γ* activation provides a potent means for reducing proinflammatory mediators after CNS injury, including trauma to the spinal cord. This is further suggested by recent SCI studies which revealed a reduction in gliosis, cytokines, and adhesion molecules [16]. Many SCI studies have demonstrated that postinjury treatment with anti-inflammatory agents results in significantly improved anatomical and functional recovery [49–54]. Thus, the anti-inflammatory actions of activating the PPAR*γ* pathway could provide another mechanism for reducing the deleterious proinflammatory cascades initiated after SCI.

## 6. DEMYELINATION OF SURVIVING AXONS AFTER SCI

Another pathological feature of acute and chronic SCI tissue is demyelination of axons that survive the initial traumatic event [55–57]. Loss of myelin will lead to conduction delays and/or frank conduction block. Because axons traversing the injury site are the sole remaining connection between the brain and caudal spinal neurons, inefficient communication through these axons is a significant clinical issue. Demyelination is due to loss of oligodendrocytes, which are killed at the injury epicenter within hours of the injury and continue to undergo apoptosis in rostral and caudal white matter for many weeks after SCI [58–60]. The potential mechanisms responsible for acute and delayed oligodendrocyte death are numerous. For instance, oligodendrocytes are known to be susceptible to glutamate excitotoxicity, which could contribute to the early loss of these cells. Oligodendrocytes and their myelin membranes are also vulnerable to lipid peroxidation, which, as stated above, is prevalent throughout the first week after injury. Lastly, proinflammatory mediators such as TNF*α* and IL-1*β* can lead to oligodendrocyte death.

Since PPAR*γ* activation can counteract many of these deleterious processes, this pathway may thereby promote oligodendrocyte survival and myelin preservation following CNS damage. Indeed, treatment with a PPAR*γ* agonist markedly improved myelination and decreased lesion area in the CNS of animals with EAE [33, 44, 48, 61]. In addition, a PPAR*γ* agonist was able to reduce myelin damage in an in vitro model of inflammatory demyelination [62]. Therefore, treatment with PPAR*γ* agonists after SCI could potentially lead to improved oligodendrocyte survival and better myelin preservation. Indeed, we have noted that when the PPAR*γ* agonist pioglitazone was given to rats after SCI, a significant increase in sparing of white matter distal to the lesions was detected [15]. This likely contributed to the improved locomotor function detected in our study and others [15, 16]. Thus, acute treatment of SCI patients with a PPAR*γ* agonist could potentially improve tissue sparing and thereby allow for a greater level of locomotor abilities as well as other important functional outcomes. For example, neurons controlling bowel and bladder function are located within the lower spinal cord, including the lumbar and sacral segments. Because most SCI's occur in more rostral segments, neuronal circuits that directly control bowel and bladder are often intact after SCI. However, significant and permanent bowel and bladder dysfunction occurs due to loss of descending signals carried by axons that are lost or demyelinated at the impact site. Therefore, enhanced preservation of myelin or promotion of remyelination at the injury site could lead to functionally significant improvements in the quality of life for SCI patients.

## 7. NEURON LOSS AFTER SCI

Contusion-type injuries are the most commonly sustained trauma to the spinal cord. Because of the high degree of vascularization and the dynamic forces encountered within the gray matter during contusive injuries [63], the lesions evolve as centralized fluid-filled cavities originating within gray matter regions at the lesion site that extend into rostral and caudal segments. Thus, even mild contusions can result in significant neuron loss over multiple segments of spinal cord. As stated above, neurons not killed by the initial impact can fall victim to secondary cascades, including ischemia, excitotoxicity, lipid peroxidation, and proinflammatory mediators. This neuron death will lead directly to loss of function in the muscles innervated by motor neurons at the segment of injury. Since the majority of injuries occur in the cervical spinal cord, SCI often means loss of function in the arms and hands. In addition, motor neurons driving respiration are found within C3–C5, so injuries that directly damage these segments frequently result in respirator dependence.

Clearly, therapies that protect neurons from secondary injury cascades after SCI are of great importance. Given the potential beneficial actions of PPAR*γ* activation discussed above, neuroprotection after SCI is an important potential therapeutic target for PPAR*γ* agonists. Indeed, improved neuronal survival following PPAR*γ* agonist treatment has already been noted in several models of CNS disorders. For instance, treatment with the PPAR*γ* agonist pioglitazone promoted motor neuron survival and increased muscle fiber diameter in a transgenic model of ALS [32]. PPAR*γ* agonists also increased neuron survival and decreased lesion sizes in models of Parkinson's disease [43, 46], central inflammation [34], intracerebral hemorrhage [38], and cerebral ischemia [5, 6, 31, 35]. These beneficial effects were likely mediated through a reduction in the indirect actions noted above, including lipid peroxidation, proinflammatory signals, and extracellular glutamate levels. However, PPAR*γ* activation may also have a direct effect on neurons. Neuronal expression of PPAR*γ* has been detected in the intact CNS and an upregulation of PPAR*γ* was observed in neurons in the ischemic penumbra following focal cerebral ischemia [5, 6, 31, 35]. Furthermore, cultured neurons treated with PPAR*γ* agonists were protected from glutamate-induced death demonstrating a direct action of PPAR*γ* activation in neurons [22]. Thus, if a PPAR*γ* agonist was delivered soon after SCI, significant neuronal sparing may be achieved which would likely translate into better functional preservation and improved quality of life for SCI patients.

## 8. SUMMARY

The PPAR pathway appears to play an important role in recovery from CNS disorders (Table 1). Indeed, several studies suggest that endogenous ligands present in the damaged CNS can activate the PPAR*γ* pathway and contribute to anatomical preservation. This is illustrated by studies demonstrating that PPAR*γ* antagonists potentiate tissue pathology after cerebral ischemia [5, 6]. Exacerbation of neuropathology also occurs when EAE is induced in the presence of a PPAR*γ* antagonist or when disease is induced in PPAR*γ* knockout mice [61, 67]. Thus, endogenous PPAR*γ* activation may be an essential component of promoting spontaneous reparative mechanisms that are initiated in the injured brain and spinal cord. This endogenous response appears submaximal, however, as the numerous studies discussed above suggest that pharmacological activation of the PPAR*γ* pathway subsequent to damage may significantly improve recovery. In the realm of SCI research, treatments are severely lacking. Therefore, manipulating the PPAR*γ* pathway appears to hold great potential as a therapy for treating human SCI.

## Figures and Tables

**Table 1:** Summary of effects mediated by PPAR*γ* activation in CNS injury or disease models.

Disease/injury model	Known PPAR*γ* effects	References
Spinal cord injury	↑locomotor recovery	[15, 16]
	↑myelin sparing	
	↑motor neuron sparing	
	↓glial activation	
	↓proinflammatory cytokines	

Experimental allergic encephalomyelitis *(model of multiple sclerosis)*	↑myelin sparing	[33, 44, 48, 61]
	↓lesion size	
	↓inflammatory cell infiltrate	
	↓proinflammatory cytokines *&* chemokines	
	↓clinical score (better recovery; lower no. of relapses)	

Amyotrophic lateral sclerosis	↑motor neuron survival	[32]
	↓glial activation	
	↓ COX-2	
	↓ iNOS	
	↑longevity, delayed disease onset	

Parkinson's disease	↑dopaminergic neuron survival	[43, 46]
	↓gliosis	
	↓ iNOS	
	↓ NF*κ*B translocation to the nucleus	
	↑ I*κ*B*α*	

Cerebral ischemia	↑neuron survival in penumbra	[6, 31, 35, 37, 64]
	↓ lesion size	
	↓COX-2	
	↓ proinflammatory cytokines	
	↑ antioxidants	

Cerebral hemorrhage	↑ catalase	[38]
	↓NF*κ*B	
	↓ neutrophil infiltration	
	↓ apoptosis	
	↓ behavioral dysfunction	

Alzheimer's disease	↓gliosis	[7, 45, 65, 66]
	↓COX-2 *&* iNOS	
	↓proinflammatory cytokines	
	↓A*β*1-42+ amyloid plaques	
	↓*β*-secretase mRNA	
	↓monocyte differentiation into macrophages	
